# The 1-Megapixel pnCCD detector for the Small Quantum Systems Instrument at the European XFEL: system and operation aspects

**DOI:** 10.1107/S1600577520015659

**Published:** 2021-01-28

**Authors:** Markus Kuster, Karim Ahmed, Kai-Erik Ballak, Cyril Danilevski, Marko Ekmedžić, Bruno Fernandes, Patrick Gessler, Robert Hartmann, Steffen Hauf, Peter Holl, Michael Meyer, Jacobo Montaño, Astrid Münnich, Yevheniy Ovcharenko, Nils Rennhack, Tonn Rüter, Daniela Rupp, Dieter Schlosser, Kiana Setoodehnia, Rüdiger Schmitt, Lothar Strüder, Rico Mayro P. Tanyag, Anatoli Ulmer, Hazem Yousef

**Affiliations:** a European XFEL, Holzkoppel 4, 22869 Schenefeld, Germany; b PNSensor GmbH, Otto-Hahn-Ring 6, 81739 München, Germany; c Max-Born-Institute, Max-Born-Straße 2A, 12489 Berlin, Germany; d LFKP, ETH Zürich, John-von-Neumann-Weg 9, 8093 Zürich, Switzerland; e IOAP, Technische Universität Berlin, Hardenbergstraße 36, 10623 Berlin, Germany

**Keywords:** X-ray detector, photon detection, pnCCD, X-ray CCD, free-electron laser

## Abstract

The X-ray free-electron lasers that became available during the last decade, like the European XFEL (EuXFEL), place high demands on their instrumentation. Especially at low photon energies below 1 keV, detectors with high sensitivity, and consequently low noise and high quantum efficiency, are required to enable facility users to fully exploit the scientific potential of the photon source. A 1-Megapixel pnCCD detector with a 1024 × 1024 pixel format has been installed and commissioned for imaging applications at the Nano-Sized Quantum System (NQS) station of the Small Quantum System (SQS) instrument at EuXFEL. The instrument is currently operating in the energy range between 0.5 and 3 keV and the NQS station is designed for investigations of the interaction of intense FEL pulses with clusters, nano-particles and small bio-molecules, by combining photo-ion and photo-electron spectroscopy with coherent diffraction imaging techniques. The core of the imaging detector is a pn-type charge coupled device (pnCCD) with a pixel pitch of 75 µm × 75 µm. Depending on the experimental scenario, the pnCCD enables imaging of single photons thanks to its very low electronic noise of 3 e^−^ and high quantum efficiency. Here an overview on the EuXFEL pnCCD detector and the results from the commissioning and first user operation at the SQS experiment in June 2019 are presented. The detailed descriptions of the detector design and capabilities, its implementation at EuXFEL both mechanically and from the controls side as well as important data correction steps aim to provide useful background for users planning and analyzing experiments at EuXFEL and may serve as a benchmark for comparing and planning future endstations at other FELs.

## Abstract 2

A description of the 1-Megapixel pnCCD detector design and capabilities, its implementation at EuXFEL’s Small Quantum Systems Instrument both mechanically and from the controls side as well as important data correction steps aim to provide useful background for users planning and analyzing experiments at EuXFEL and may serve as a benchmark for comparing and planning future endstations at other FELs.

## 1. Introduction   

The increasing availability of X-ray free-electron laser (FEL) sources (Altarelli *et al.*, 2007 ▸; Emma *et al.*, 2010 ▸; Gatner, 2010 ▸; Ishikawa *et al.*, 2012 ▸; Kang *et al.*, 2017 ▸) over the last decade has triggered various new developments and improvements in spectrometer and detector technologies in order to enable the scientific community to fully exploit the capabilities of these sources for experimental investigations.

In the area of detector technology, silicon-based direct detection hybrid pixel detectors (HPDs) with up to 4.5 MHz imaging capability became available during the last years, mainly enabled by the rapid advancement in microelectronics technology. A few examples are integrating detectors like the Large Pixel Detector (LPD) (Hart *et al.*, 2012 ▸), the Adaptive Gain Integrating Pixel Detector (AGIPD) (Allahgholi *et al.*, 2019 ▸) and the DEPFET Sensor with Signal Compression (DSSC) (Porro *et al.*, 2012 ▸). Being the result of a more than ten years long development effort, these technologies have the potential to open new avenues for scientific applications at FELs.

In particular, scientific applications relying on very low photon detection noise and/or spectro-imaging capability benefit from the continuous improvements of the charge coupled device (CCD) technology during the last decade. The FastCCD (Doering *et al.*, 2012 ▸), MPCCD (Kameshima *et al.*, 2014 ▸) and pnCCD (Strüder *et al.*, 2010 ▸) are a few exemplary monolithic detectors playing an important role at storage rings and FELs [see Graafsma *et al.* (2020 ▸) and Hatsui & Graafsma (2015 ▸) for an in-depth review].

The European XFEL started user experiments in 2017 (Decking *et al.*, 2020 ▸) and is operating in the soft X-ray and hard X-ray regime, *i.e.* at photon energies from 270 eV up to more than 20 keV. Presently six scientific instruments (Tschentscher *et al.*, 2017 ▸) are providing high-performance experimental tools for a broad range of scientific applications in research fields like fundamental research on atoms and molecules, chemistry, solid state physics and biology.

One of these instruments, the Small Quantum Systems (SQS) instrument, is dedicated to investigations of non-linear and time-resolved phenomena on atomic and molecular systems, as well as to studies of clusters and nano-objects under irradiation with ultra-short, highly intense soft X-ray pulses. The SQS scientific instrument is located behind the SASE 3 soft X-ray undulator, which delivers intense, ultra-short (about 25 fs) and spatially coherent pulses of X-ray light, currently with photon energies in the range from 0.5 to 3.0 keV with smooth wavelength tunability and pulse energies up to 5 mJ.

**Figure 1:**
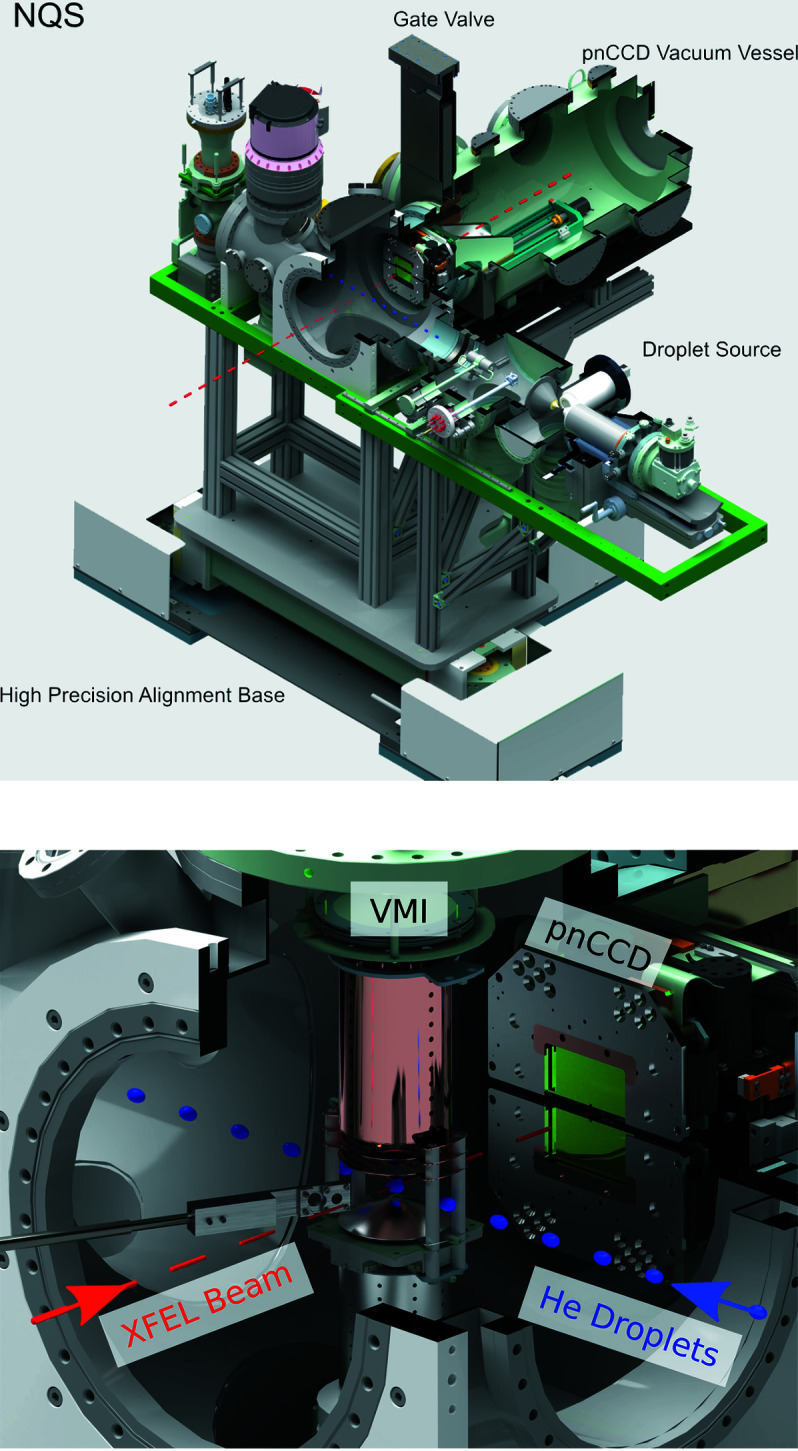
Top: overview of the NQS experimental chamber at the SQS instrument. Bottom: enlarged view of the interaction region showing the pnCCD detector mounted in the direction of the photon beam. The photon beam enters the NQS chamber from the bottom left (red dashed line) and the droplet source injects the sample from the bottom right (blue droplets).

Three versatile and interchangeable experimental stations are available at the SQS instruments for the user community. One of them, the Nano-sized Quantum Systems (NQS) station (see Fig. 1 ▸), is designed for investigations of the interaction of intense FEL pulses with clusters, nano-particles and small bio-molecules by combining photo-ion and photo-electron spectroscopy with coherent diffraction scattering techniques. Experiments based on electron and ion spectroscopy are realized by using velocity-map imaging (VMI) (Eppink & Parker, 1997 ▸) and time-of-flight (TOF) mass spectrometers (Wiley & McLaren, 1955 ▸), respectively. For experiments on coherent diffraction imaging a 1-Megapixel pnCCD detector has been available since June 2019.

## 2. The pnCCD camera system   

The pnCCD technology was originally developed for spectro-imaging applications in X-ray astronomy (Strüder *et al.*, 1990 ▸, 2001 ▸) and has been used in this field for more than 20 years (Leibniz Institute for Astrophysics, 2020 ▸). Since then, pnCCDs have been successfully deployed for a broad range of scientific applications with significantly different scientific requirements. This includes imaging of electrons (Ryll *et al.*, 2016 ▸), as well as of photons from visible light to X-rays (Strüder *et al.*, 2010 ▸), soft gamma ray energies and indirect detection of dark matter (Kuster *et al.*, 2007 ▸). Depending on the experimental scenario, imaging of single photons is combined with high-resolution spectroscopy.

The design of the 1-Megapixel pnCCD camera system for the SQS instrument is optimized to fulfill the technical and scientific requirements of the NQS endstation, while maintaining the flexibility to move the camera as a self-contained unit between different experiments at the European XFEL. The core component of the detector is a fully depleted pnCCD. A valuable feature of this kind of sensor is its integrated front-end electronics and fully column parallel readout providing excellent imaging and spectroscopic performance, long-term stability and high-speed readout.

### 2.1. Detector mechanics, vacuum and cooling system   

Figure 1 ▸ shows a schematic cross section of the pnCCD detector system with its subcomponents as implemented at the NQS endstation. The pnCCD detector is mounted in the beam direction at a distance of about 30 cm behind the interaction volume defined by the crossing of the FEL (dashed red line) and the sample delivery axis (blue dotted line) (Fig. 1 ▸). The FEL beam is focused down to a spot size of about 1.5 µm × 1.5 µm (FWHM) by a pair of highly polished Kirkpatrick–Baez (KB) mirrors. The NQS chamber is kept at a vacuum level of 10^−8^ to 10^−9^ mbar in order to operate the electron and ion spectrometer. A set of apertures is installed at the entrance of the chamber allowing to define the FEL beam profile and to reduce stray light on the photon detector.

A DN 300 shutter connecting the pnCCD camera’s vacuum chamber to the NQS endstation can be closed to seal it off hermetically, if either the detector system is operated stand-alone (*e.g.* for system qualification and calibration) or alignment procedures of the target beam are carried out that may bear a risk for the pnCCD detector. The design of the ultra-high vacuum system of the camera and detector components enables operation in the low 10^−8^ mbar pressure range.

The pnCCD sensor plane and its cooling infrastructure are mounted on a 300 mm-long and UHV-compatible linear stage, enabling the flexibility of changing the distance between the sample and the sensor plane along the FEL beam axis [*i.e.* in the *z*-direction when referring to beamline coordinates, see Fig. 1 ▸ and Sinn (2013 ▸)]. In the fully retracted position, the sensor plane is at a distance of 350 mm to the beam focus. At the closest distance to the beam focus, *i.e.* at a distance of 50 mm, the sensor plane sticks out of detector’s vacuum chamber into the NQS chamber. Positioned to those most extreme positions, the sensor plane covers scattering angles of up to 9.5° and 49.4°, respectively.

**Figure 2:**
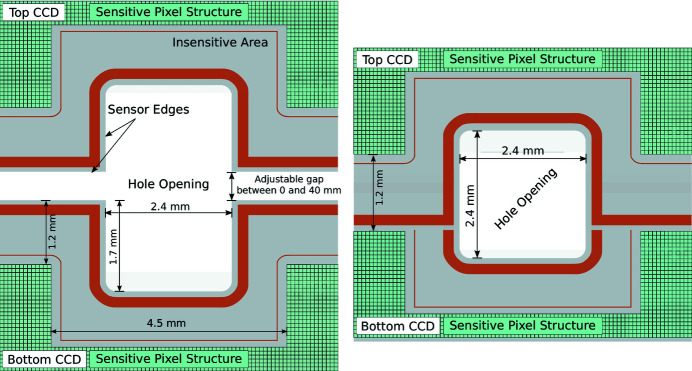
Schematic view of the sensor highlighting the region close to the central rectangular hole. The sensor and hole geometry are shown for two configurations. Left: the top and bottom CCD are moved up and down into an open hole configuration. Right: the smallest hole configuration is shown. Due to the staggered arrangement of the upper and lower CCD, the upper CCD is overlapping the lower CCD such that the slit is completely closed and a 2.4 mm × 2.4 mm large quadratic hole remains in the center of the sensor plane.

Two vacuum motion stages allow separation of the sensor halves in the vertical (*y*) direction with a precision of 1 µm, *i.e.* perpendicular to the FEL beam. The resulting gap between the two halves of the pnCCD sensor is up to 40 mm wide, allowing the user to enlarge the detection plane in order to cover a larger *q* range. Passage of the primary FEL beam is enabled by a laser-cut 2.4 mm × 2.4 mm large rectangular hole as illustrated in Fig. 2 ▸. The slit and the hole can be completely closed, as the two sensor planes can be staggered leaving a gap of 3 mm between the sensor planes in the beam direction, with the top half covering the lower half. When the slit is completely closed, a 1.2 mm insensitive area remains between the lowest pixel column of the top detector half and the uppermost pixel column of the bottom half, whereby no part of the sensitive sensor area is blocked.

The significantly reduced thermally generated dark current of the latest generation of pnCCDs, in comparison with earlier versions (Holl *et al.*, 2006 ▸), allows operating the pnCCD sensors at temperatures up to −20°C while achieving an electronic noise level of 3 e^−^ r.m.s. in high gain and about 10 e^−^ r.m.s. in low gain. To achieve the best imaging and spectroscopic performance, a temperature stability of the sensor better than ±1 K is required.

**Figure 3:**
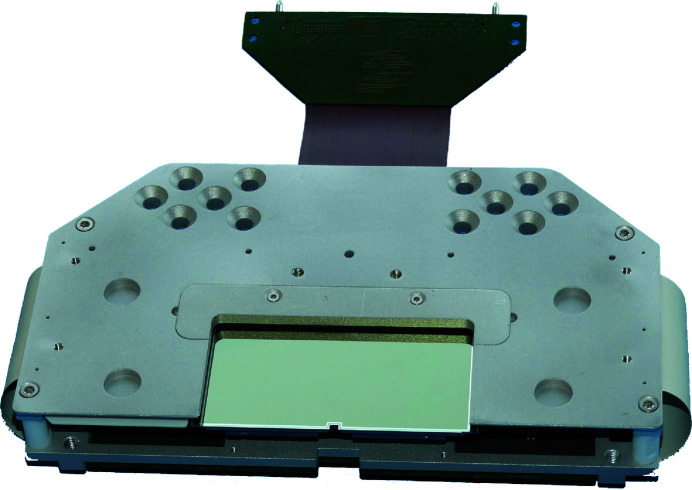
Image of the pnCCD sensor hybrid board. Two of these boards are installed in the 1-Mpix camera. Each board carries one sensor half, 512 × 1024 pixels large and the in vacuum readout electronics. The flex lead shown on the top of the image, provides the electronic interface to the part of the readout electronics installed outside the vacuum chamber.

**Figure 4:**
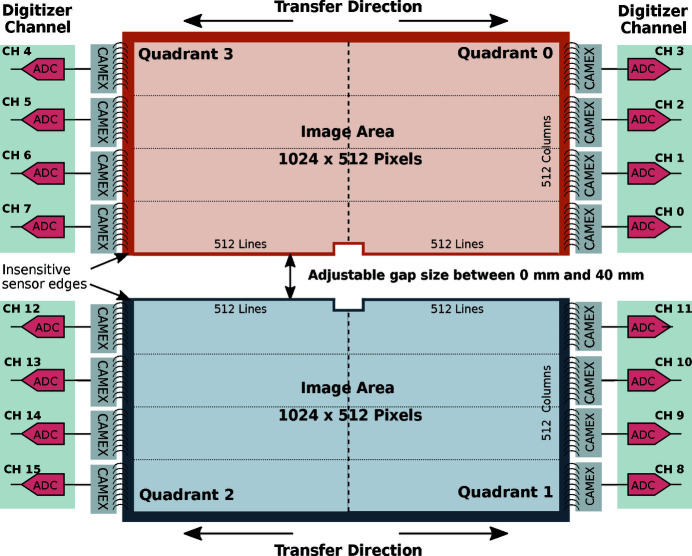
Schematic view of the pnCCD sensor geometry as seen when looking in the beam direction onto the detector (in beamline coordinates this corresponds to the positive *z* direction), *i.e.* the down-stream FEL beam direction. The sensor is divided into two halves, which can be operated independently. The designation of the digital signal channels and detector quadrants is the same as the one used in the metadata of the Karabo online data stream and the HDF5 raw/calibrated data files. Both data sources are accessible to beamline users for data analysis.

The thermal power budget of the sensor plane is dominated by the thermal dissipation power of the 16 Application Specific Integrated Circuit (ASIC) chips mounted next to the pnCCD sensors on the sensor hybrid board (see Section 3, Fig. 3 ▸ and Fig. 4 ▸). During readout of the pnCCD, each of the ASICs contributes with 0.3 W to the total power budget of 5 W. In order to reduce the power dissipation to a minimum, the ASICs are switched to stand-by when not needed for signal processing, *e.g.* during the integration phase of the CCD’s image-taking cycle.

The detector’s active cooling system is optimized to the above-mentioned requirements. It is seamlessly integrated into the camera and the motion system. Rigid high-conductivity copper connections transfer the heat from the Al–Si alloy sensor frame to the rear side of the sensor plane. Subsequently, four flexible copper braids transmit the dissipated thermal power to the in-vacuum cold fingers of two Polycold^®^ Compact Coolers (PCCs). In the configuration used for this application, the PCCs provide a maximum cooling power of ∼24 W. With this design, the sensor temperature is stable to better than ± 0.1 K.

This temperature tolerance is sufficient in significantly minimizing the detector’s dark current and noise, as needed in photon-sensitive X-ray imaging. The time required to cool the pnCCDs in vacuum from room temperature to the nominal operating temperature of −30°C and to reach stable thermal operating conditions is about 3 h. Temperature stability over a time scale of days within the above-mentioned requirement has been demonstrated with this design.

During detector operation, the temperature of the pnCCD sensor is continuously monitored via four platinum resistance thermometers (type PT 1000) mounted on the cold fingers and the copper block attached to the pnCCD modules. The temperature readings, amongst other slow control values, are logged by the Karabo control system of the European XFEL (Hauf *et al.*, 2019 ▸). The pnCCD calibration pipeline can use these temperature values as reference for data correction and processing (see Section 3).

### 2.2. pnCCD sensor and signal processing   

The pnCCD sensor plane has a sensitive area of 7.68 cm × 7.68 cm and consists of two monolithic chips (sensor halves) as schematically shown in Fig. 4 ▸. Both are identically designed back-illuminated pnCCDs. Each has 1024 × 512 pixels and a fully depleted, 450 µm-thick sensitive volume. The pixel size of 75 µm × 75 µm allows for sub-pixel position resolution of better than 10 µm (at normal incidence) in the single photon detection regime when charge centroiding techniques are applied (Ihle *et al.*, 2017 ▸). The two halves are operated and read out independently in a split-frame mode (see Fig. 4 ▸). Further performance parameters of the sensor and readout electronics are summarized in Table 1 ▸.

During the readout process, the signal charges generated by X-ray photons are transferred line by line along the pnCCD transfer columns. Each column is terminated by a charge-collecting anode connected to an integrated n-channel junction gate field-effect transistor (JFET). This on-chip amplifier is operated in source follower mode and bonded to one input channel of the CMOS Amplifier and MultiplEX (CAMEX) ASIC (Buttler *et al.*, 1988 ▸; Herrmann *et al.*, 2008 ▸). This column-parallel signal amplification concept allows for high frame rates in combination with low noise performance.

In total eight CAMEX ASICs with 128 inputs each are required for handling the signal of the 1024 JFET source follower outputs of each pnCCD chip. For further signal processing, the CAMEX provides two-stage amplification, filtering, noise bandwidth limitation and eight-fold correlated double sampling (CDS). The multiple baseline and signal sampling enabled by the CDS increases the signal-to-noise ratio proportional to 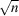, where *n* is the number of sampling points. Through a digital control register it is possible to adjust the gain of the pre-amplifiers to the experimental conditions. Apart from the highest gain *g* = 1, gain values of *g* = 1/4, 1/16, 1/64, 1/256 and 1/1024 can be configured before data taking. Finally the signal is stored in a sample-and-hold (S&H) stage before all 128 parallel channels are multiplexed in one analog output channel per CAMEX.

After serialization by the CAMEX, the analog signal is transferred to and digitized by two SIS8300 µTCA digitizer boards providing ten ADC channels with 16 bit resolution each. The EuXFEL uses these boards as a facility-wide standard for high-speed digitizer applications. The boards sample the signal with a frequency between 10 MHz and 125 MHz per channel. Application-specific readout, data formatting, data reduction and processing algorithms can be implemented on a Virtex V field programmable gate array (FPGA) available on the SIS8300 board (Struck Innovative Systems, 2020 ▸). Each board in addition reads the pixel clock signal provided by the timing sequencer of the pnCCD for synchronization. The digitizer FPGA firmware is configured to sample 800 kSamples per CAMEX and image, as determined by the 10 Hz train trigger, which corresponds to one or two samples per pixel and readout cycle. The CAMEX is typically operated at 10 MHz, enabling image rates up to 100 frames per second.

Figure 4 ▸ shows the geometric arrangement of the sensor plane, the signal transfer direction, and illustrates how the ASIC chips are arranged on the right and left side of the sensor chips. The designation of the output channels of the ADCs and detector quadrants is the same as the one used in the metadata of the Karabo online data stream and the HDF5 (The HDF Group, 1997–2020 ▸) raw/calibrated data files.

Visible and infrared background light, *e.g.* from a 800 nm pump-laser, can significantly deteriorate the signal-to-noise ratio of an experiment, especially when aiming at photon energies <1 keV and small scattering signals. A blocking filter for visible and IR light is therefore crucial for maintaining a high signal-to-noise ratio in the presence of background signal sources. In general, two technological solutions exist to mitigate this problem, either by using a thin and coated foil, mounted in front of the sensor plane, or by a filter deposited on the entrance window of the sensor. Appropriate foils, having a thickness of a few hundred nanometres, are very fragile and require a support structure, that may additionally scatter photons as undesired background signal.

**Figure 5:**
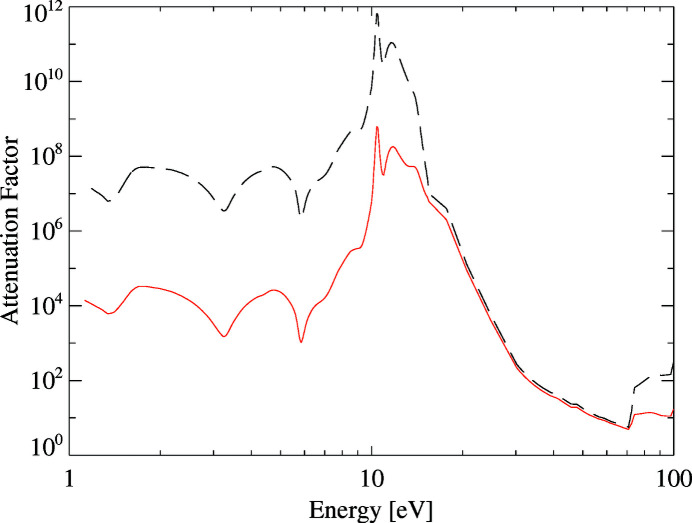
The attenuation factor of a 150 nm (black dashed line) and 60 nm (red line) thick aluminium, SiO_2_ and Si_3_N_4_ light-blocking filter deposited on the entrance window of the pnCCD sensor. The filter efficiently suppresses near-infrared, visible and ultaviolet radiation up to 10 eV by a factor 10^7^ or 10^4^, respectively. The influence of the aluminium absorption decreases due to the plasma frequency cutoff at ω_p_ ≃ 16 eV resulting in the refractive index *n* becoming increasingly smaller between 15 eV and 73 eV, the Al *L*-edge.

**Figure 6:**
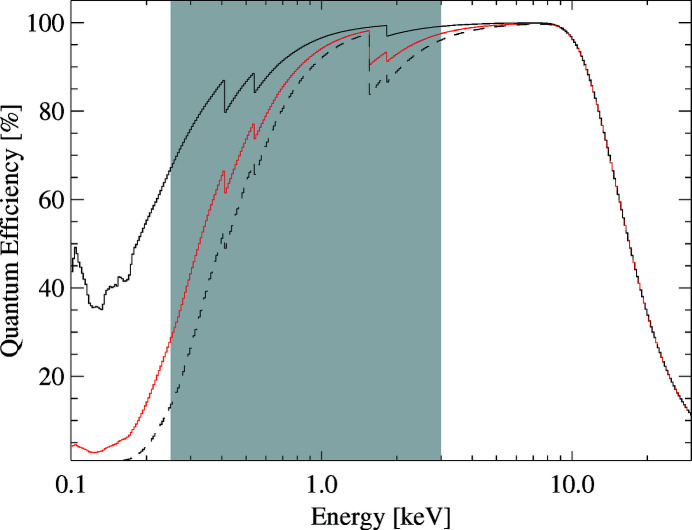
The quantum efficiency of the pnCCD sensor in the energy range between 100 eV and 30 keV is shown. The black dashed line and the red solid line correspond to the quantum efficiency of a pnCCD sensor with a 150 nm and 60 nm thick aluminium entrance window, respectively. For comparison, the quantum efficiency of an entrance window without an aluminium layer is shown (solid black line). Note that in the current installation an Al thickness of 150 nm is applied, while a future upgrade is planned with a 60 nm thick Al coating. The gray area marks the baseline photon energy available at the SQS instrument.

With a light-blocking filter directly deposited on the entrance window of the sensor, we can circumvent these disadvantages. The design of such a filter requires a careful trade-off between attenuation of visible light and the desired X-ray quantum efficiency. For this detector, we have chosen an in total 150 nm thin blocking filter, consisting of layers of aluminium, SiO_2_ and Si_3_N_4_ directly deposited on the p+ doped silicon sensor backside. During a major upgrade planned in the second half of 2020, we implemented a new sensor module with an improved sensitivity for low-energy photons, enabled by the implementation of a thinner aluminium entrance window with a thickness of 60 nm. The combination of the three layers efficiently attenuates photons with energies between 1 eV and 10 eV by at least a factor of ∼10^4^ using a 60 nm and ∼10^7^ with a 150 nm thick aluminium layer as shown in Fig. 5 ▸. A further benefit of this kind of ultra-thin entrance window is its homogeneous response to low-energy photons, resulting in good energy resolution and high quantum efficiency of at least 75% in the energy range between 0.6 keV and 14 keV (see Fig. 6 ▸).

### 2.3. pnCCD readout timing and synchronization to the XFEL pulse pattern   

**Figure 7:**
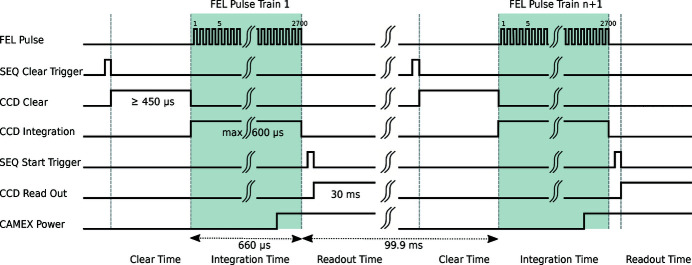
Schematic diagram illustrating the pnCCD readout timing and synchronization to the EuXFEL pulse pattern. For a more detailed description, we refer the reader to the text.

The EuXFEL machine delivers up to 27000 spatially coherent X-ray pulses per second in a unique time structure. Ten times per second, a train of equidistantly separated X-ray pulses arrives at the sample interaction point in the NQS sample chamber as shown in the topmost line of Fig. 7 ▸. Each pulse train can contain between 1 and up to 2700 X-ray pulses with a minimum inter pulse separation of 220 ns. This corresponds to a maximum pulse repetition frequency of 4.5 MHz.

The synchronization of the detector is accomplished through the EuXFEL timing system (Gessler *et al.*, 2020 ▸), which provides triggers to the sequencer of the pnCCD. Since the sequencer uses an internal clock, the SIS8300 ADC is synchronized to the sequencer to ensure phase aligned readout of the detector signals. In this way, the detector front-end is correctly aligned with the timing of the machine.

Since the pnCCD is continuously biased, the sensor bulk is sensitive to visible, IR and X-ray photons at any time of the integration and readout cycle. As a consequence, photons being absorbed in the depleted sensor bulk between two consecutive readout cycles would create a potential background signal which does not originate in an interaction of the FEL beam with the sample. Therefore, the pnCCD sensor is cleared of charge before the arrival of the first X-ray FEL pulses by quickly shifting residual charges to the readout anode and discarding the charge signal.

This 420 µs-long clear process is followed by the signal integration period (see Fig. 7 ▸) with a duration chosen according to scientific requirements. Its minimum duration is 600 ns, with an upper limit given, for example, by the desired time resolution. A typical value is 600 µs which is sufficient to record all signals produced by the X-ray pulses within one pulse train. The final signal readout, amplification, filtering and digitization take an additional 14 ms. The readout sequence is synchronized to the EuXFEL bunch pattern through the 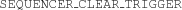 and 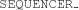 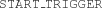 signals provided by the C&C system, respectively. Both trigger signals are sent to Karabo.

In general the pnCCD’s sequencer generating the readout sequence and control signals for the CAMEX and the CCD can be programmed in a very flexible way, such that the CCD’s readout and signal processing sequence can be adjusted to and optimized for specific experimental requirements. Nevertheless, for most scenarios, changing the readout sequence requires a recalibration of the detector for achieving the ideal performance as shown in Section 4.

## 3. Data processing, correction and calibration   

The pnCCD online data processing and correction is integrated into Karabo as configurable pipelines built of different components, referred to as devices. They provide specific signal correction, data processing, data managing and data handling functionality.

Both the digitized analog and pixel clock signals are read out from the SIS8300 boards by a C++ software integrated as a device into the control system. A second device samples the pixel signal synchronized to the pixel clock (see Fig. 7 ▸). Furthermore, it reformats the one-dimensional digitizer data stream into 16 sub-images with 128 × 512 pixels each (one per CAMEX output channel) and assembles them into the full megapixel image. Since the two digitizer cards are read out independently, the two input data streams are synchronized by the timing information provided as train IDs in the trigger process. The assembled image data are then output as a 2D array with 16 bits resolution per pixel via a 10 Gbit network link to the data acquisition system. Finally, the full 2D images are stored into HDF5 files, and provided as a data stream to online visualization and processing. All software discussed are running on a CPU card hosted in the same µTCA crate with the SIS8300 boards, communication being done via PCI Express.

As offline data processing is not required for rapid feedback and direct interfacing to detector hardware, it is conceptually implemented as a stand-alone processing pipeline. It is aimed at providing the highest possible data quality, *i.e.* hardware-limited calibrated images and spectroscopic data. This pipeline is implemented in Python and distributed through the pyDet­Lib library. On the other hand, online processing and visualization is required for rapid feedback to the detector operators and users and, therefore, another set of processing pipelines.

Calibration and correction parameters and their metadata are managed by the XFEL calibration database, stored in HDF5 files and archived on tape. Parameters used for data correction are selected by evaluating detector settings and conditions under which the data for correction and calibration have been acquired. The correction parameter set closest in time to the measured scientific data to be corrected and best matching the detector settings is finally used. The parameter sets are divided into two different classes, one set is provided by the facility and the other set is acquired during the user beam time. The latter parameter set might depend on and change according to experiment conditions, while the first parameter set does not change significantly in the long term (*i.e.* on a time scale of weeks to months).

For the pnCCD, the following calibration and correction parameters are available for each gain setting through the XFEL calibration database and, if necessary, may be updated before a user’s beam time (Kuster *et al.*, 2014 ▸):

(i) The gain vectors *g*_1_, *g*_1/4_, *g*_1/16_, *g*_1/64_, *g*_1/256_ and *g*_1/1024_ for conversion of analog digital units (ADU) to photon numbers;

(ii) A vector characterizing the relative variation of the amplification *g*_rel *i*,*k*_ between the 128 different parallel CAMEX readout channels for all 16 ASICs; and

(iii) The charge transfer inefficiency (CTI) for each sensor column CTI_*i*_ quantifying the charge loss during charge transfer from the photon interaction point to the readout anode.

The following parameters are experiment specific and are determined from data acquired during an experiment:

(i) The offset map *O*_*i*,*k*_ providing the per pixel offset.

(ii) The noise map providing the per pixel r.m.s. noise *N*_*i*,*k*_, and the corresponding bad pixel map *B*_*i*,*k*_.

In the following sections we describe the data processing steps applied to pnCCD data, as implemented in the online and offline data processing pipelines in more detail. The information provided reflects the status as of December 2019, corresponding to the following software releases: Karabo V2.4.2, pnCCD calibration pipeline pycalibration V2.0, calPy V1.9.1–2.4.1, calibration database cal_db_interactive V1.0, the fasterADC V2.6.1–2.4.0 and cppPnCCDFormatter V1.1.1–2.4.1 devices.

### 3.1. Online processing   

Before scientific data are processed online, a data set for offset and noise calculation is typically acquired before each experimental run. The offset and noise maps are subsequently calculated per pixel in one single pass using the numerical algorithm of Welford (1962 ▸). Welford’s algorithm is based on updating the sum of squares of differences,
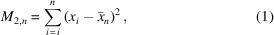
for estimating the mean of a population of *n* values. The mean *M*_2,*n*_ for the current *n* follows for *n* > 1 from 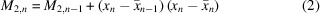
and the variance from 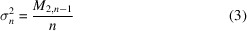
where 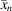 is the mean calculated from the first *n* samples. We calculate the mean signal value of each pixel *i*,*k* according to equation (2) and assign it to the offset map as value *O*_*i*,*k*_, where *i* and *k* indicate the column and line numbers of the sensor, *x*_*n*_ = *S*_*i*,*k*_ the signal charge measured at pixel position *i*,*k* and *n* the number of image frames used for the calculation. In a similar fashion, the noise map entry *N*_*i*,*k*_ is calculated from the per pixel variance as 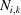 = 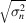. By default, the sample size for mean, variance and standard deviation calculation for each pixel is set to *n* = 500 images. The resulting offset and noise maps are finally injected into the calibration database. Either this offset map is subtracted from the scientific data during online processing or an offset map matching the experimental and detector conditions closest is used.

In the latter case, the following detector parameters are taken into consideration for selecting the best matching offset map: CCD temperature, CCD bias voltage, integration time and the chosen CAMEX gain settings. The result of the correction is stored as pixel arrays in the corrected data files. During this step, the scientific data are up-converted from 16 bit unsigned integer values to 32 bit float values.

Subsequently, a line-by-line common mode correction reduces baseline variations of the analog pixel signal. The number of pixels *N* in each line taken into account for the common mode calculation is configurable to the number of pixels per ASIC (*i.e.* *N* = 128 pixels), the dimension of a sensor quadrant (*N* = 512 pixels) or sensor half (*N* = 1024 pixels). The common mode value is then calculated from the sorted vector *S*_*k*,*i*_ as 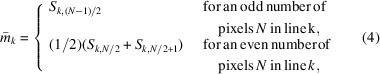
and subsequently subtracted from the signal in each pixel of one line.

For the pnCCD it has been found that evaluating the common mode as a thresholded median for each quadrant gives a good trade-off between accuracy and stability against event-rich images. In that case, only pixels in one line which are carrying a signal below the threshold *E*_th_ are taken into consideration for the calculation of the line’s median value.

Further and higher-order data treatment like charge sharing, charge transfer efficiency corrections and correction of the variations of the relative amplification of the 128 CAMEX channels are not taken into account during online processing.

The online corrected images are then passed on to the Karabo graphical user interface (Karabo GUI) for visualization or to the Karabo bridge for interfacing to user-provided processing using the ZMQ protocol (Fangohr *et al.*, 2018 ▸).

### 3.2. Offline processing   

Offline processing is performed on recorded data files. It is either automatically triggered when users migrate data to be archived and made available on the Maxwell HPC cluster (DESY, 2020 ▸), or explicitly using the Metadata Catalog web interface. Offline processing retrieves necessary calibration and correction parameters from the calibration database in a similar way as the online processing pipeline.

By default, the offset correction is always performed using the same offset maps as the ones used by the online processing pipeline. Furthermore, a line-by-line common mode correction using by default the same feature size as for online processing is applied. The result of this correction is output into the 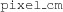 array in the corrected data files. For correcting the data presented in Section 4, a median value which is calculated from the *N* = 200 outermost pixels in each line gives the best results with respect to flat field baseline homogeneity. Attention should be paid to the fact that the common mode correction is prone to be biased or can even completely fail for very event-rich images. Consequently, the methodology for common mode correction should be carefully chosen during offline processing.

Noisy pixels, pixels providing no signal (*e.g.* the area of the central hole) or a non-physical signal and events produced by cosmic rays are excluded from the analysis and marked in the bad pixel map in the calibration data base. Pixels marked as bad are excluded from further treatment during the offline processing.

Split event correction, also called charge sharing correction or charge clustering, is always performed. It classifies clustered signals of discrete photon events by the number of adjacent pixels the charge is detected in. The signal in each pixel is probed against two thresholds prior to event classification: a primary event threshold to identify the pixel carrying the majority charge of the total signal of a photon event and a secondary (lower) threshold for identifying neighboring pixels as having registered a signal above the noise floor. Both thresholds are given in units of σ, where σ is the pixels specific r.m.s. noise given by the noise map. The algorithm then searches for valid charge clusters that can originate in a photon interaction and sums all charges above noise into an event reconstructed primary pixel carrying the majority charge. Clusters that cannot be attributed to a single photon event are identified via a special pattern index. Their charge is not summed up. The result of this correction is stored into the 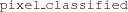 array. Additionally, a 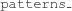 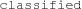 array is generated, which encodes event multiplicity and orientation.

If relative gain correction is activated, the aforementioned pixel arrays will be stored corrected for the relative difference of the amplification of the amplifiers of the 128 channels of each CAMEX.

The charge transfer inefficiency (CTI) of the present generation pnCCDs is better than ∼5 × 10^−5^, leaving a potential signal shift by 2.5% after 512 charge transfer steps. The CAMEX channel-to-channel amplification variation is less than ±5% of the detected signal charge. Both corrections are of minor importance for imaging applications and are therefore presently neglected during default offline image correction, but can be enabled during manual data reprocessing to achieve the best possible spectroscopic performance.

Conversion from analog digital units (ADU) to photon numbers is performed by means of a gain calibration vector, one for each possible gain configuration *g* = 1, 1/4, 1/16,…, 1/1024. This conversion is performed for the three output arrays: 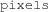, 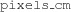 and 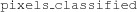.

For an in-depth description of data correction and treatment and its implementation of the XFEL pyDetLib, we refer to the corresponding user manuals (EuXFEL, 2020 ▸). The data presented in the following sections have been treated offline in the same way here in Section 3.

## 4. Detector performance   

After installation of the detector at the NQS station, we commissioned the pnCCD detector and characterized its performance before the first user beam time, which took place shortly thereafter during EuXFEL’s user runs in the first and second half of 2019 (run period 03 and 04 at EuXFEL). We investigated the detector’s noise behavior and photon response under nominal operating conditions, *i.e.* at stable sensor temperature and optimized detector operating parameters, like bias voltage settings, CAMEX configuration and readout sequence of the CCD. The performance figures provided in this section refer to the performance as measured at the SQS experimental environment.

**Figure 8:**
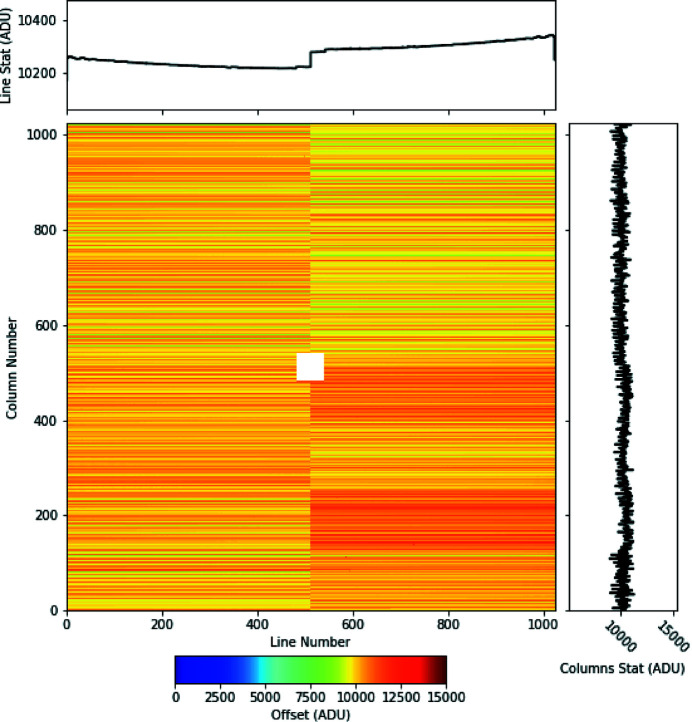
The pixel dark signal as measured at the SQS beamline during the first user operation period in 1/16 gain mode. The dark map is calculated from 400 dark frames as described in the text. The orientation of the image is the same as illustrated in Fig. 4 ▸, *i.e.* the photon signal is shifted and read out towards the CAMEX ASICs located on the left and right side of the sensor. The top and bottom histograms show the offset averaged along the CCD lines (top) and columns (right).

Figure 8 ▸ shows an exemplary offset map calculated from 400 individual images acquired with gain 1/16 during the user run period 03. The dark distribution shows the typical regular stripe structure along the sensor columns expected from a CCD with column parallel readout. This structure can be attributed to channel-to-channel baseline variations of the 128 CAMEX input channels.

**Figure 9:**
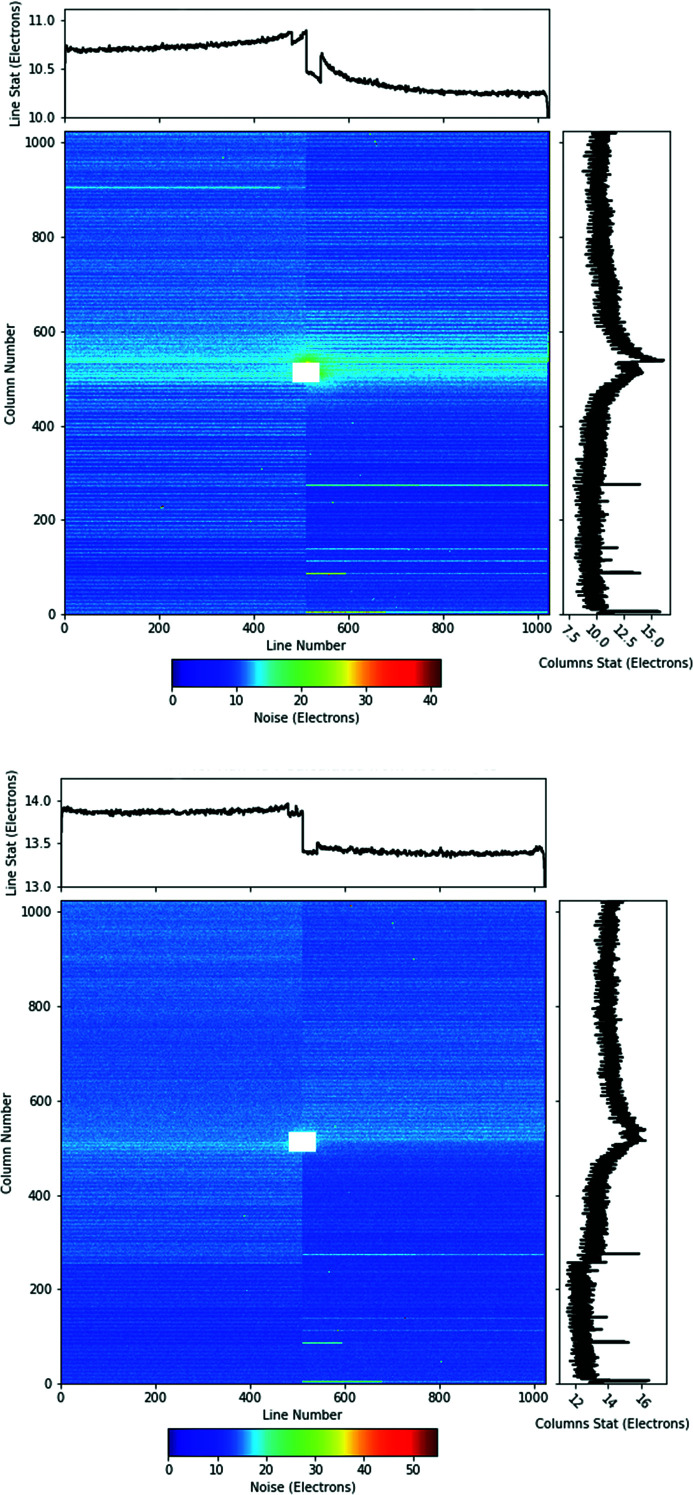
The per pixel r.m.s. noise as measured at the SQS beamline. Top: the per pixel noise measured with gain 1. Bottom: the per pixel noise measured with gain 1/16 for comparison.

The spatial distribution of the corresponding r.m.s. noise is shown in Fig. 9 ▸ measured with gain 1 (top image) and 1/16 (bottom image).

The features of the dark and noise maps in Fig. 9 ▸ can be regarded as exemplary for the operating conditions and parameter settings used with the pnCCD detector during the EuXFEL’s user beam time.

As mean dark signal calculated across the whole chip we find 10968.7 ADU. The corresponding per pixel noise of 126.15 ADU = 37.9 eV translates into an equivalent noise charge (ENC) of 10.5 e^−^, when taking the ADU to photon energy conversion factor of 0.30 eV ADU^−1^ (gain 1) into consideration. The EuXFEL pnCCD detector is thus capable of discriminating single photons with an energy of 250 eV and 500 eV from the noise floor with a significance of 6.6σ and 13.2σ, respectively.

**Figure 10:**
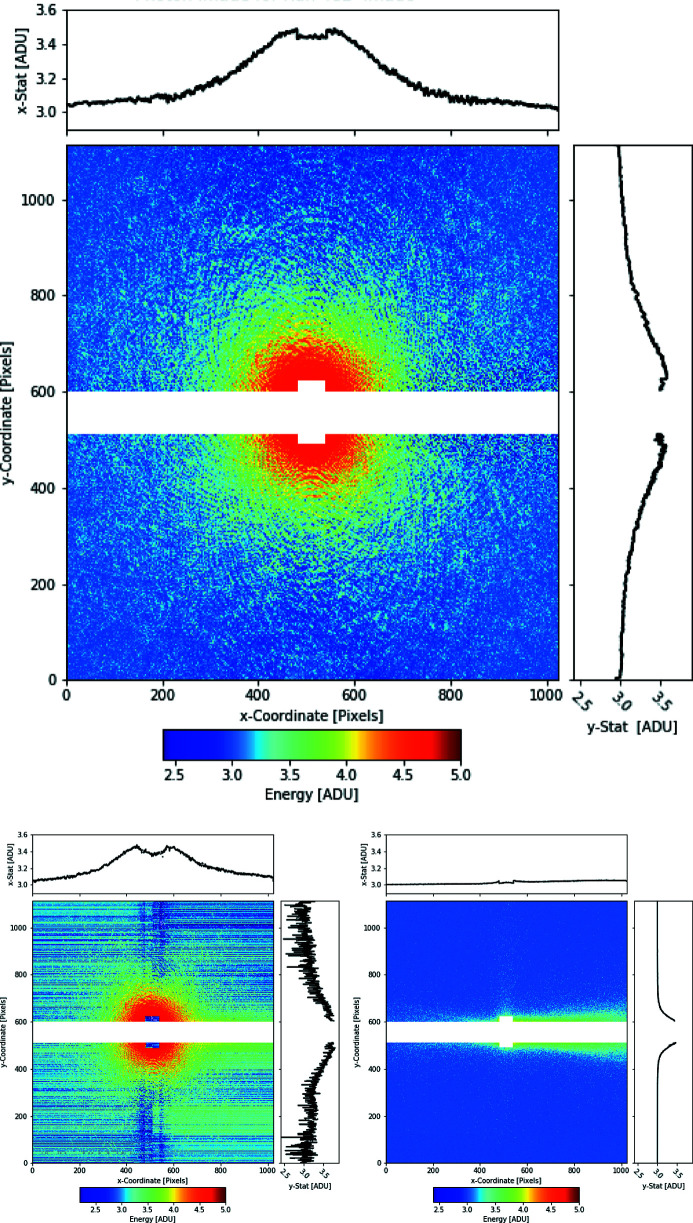
Top: fully corrected single-shot coherent X-ray diffraction image of a superfluid helium droplet doped with acetonitrile molecules. The diffraction image was obtained from a single FEL pulse at a photon energy of 1 keV and was recorded in June 2019 with the newly commissioned pnCCD sensor at the NQS chamber. During data acquisition, the detector’s gain was set to 1/16 and the top and bottom CCD modules were separated by 6.8 mm vertically. Bottom left: the uncorrected raw image in units of ADU as provided by the output of the ADC is shown. Bottom right: experiment background image obtained from a single-shot, acquired directly before the signal image.

An example of the detector’s imaging performance is demonstrated in Fig. 10 ▸. This diffraction pattern was acquired during the first user beam time using the pnCCD installed at the NQS chamber in June 2019 (Tanyag & Rupp, 2020 ▸). The diffraction image was obtained from micrometre-sized superfluid helium droplets containing millions of acetonitrile molecules. The droplets were hit by one single FEL pulse focused to a diameter of about 1.5 µm, with a photon energy of 1 keV.

The detector was configured to gain 1/16, in order to avoid saturation of the pixels close to the central imaging area. The area of the central hole is excluded from the analysis. Further data treatment includes subtracting the experimental background captured by the imaging frame before the helium diffraction pattern (bottom left picture of Fig. 10 ▸).

The influence of the data corrections applied during offline analysis is illustrated by comparing the bottom left picture of Fig. 10 ▸ with the fully corrected diffraction pattern on the top. The bottom left picture shows the same diffraction pattern as provided by the front-end electronics at the output of the ADC. Apart from normalization of the mean baseline to the baseline of the corrected image, no further data corrections were applied to this image. The uncorrected image shows all features of the detector’s dark signal, common mode effects, experiment background and the diffraction signal on top. The horizontal stripes result from variations in the baseline and gain for each CAMEX readout channel. Furthermore, a non-zero signal is apparent from the area of the central hole. This is the consequence of the way the pnCCD sensor is read out. Since the readout sequence always considers 512 pixels to be processed by the CAMEX, the CAMEX provides an output signal to the ADC even for the area corresponding to the central hole.

## 5. Conclusions and outlook   

The EuXFEL 1-Megapixel pnCCD detector enables experimental techniques like coherent diffraction imaging at the SQS instrument with single photon sensitivity on a 6.6σ and 13.2σ significance level at the lowest photon energies provided by the FEL, *i.e.* 250 eV in the future and presently 500 eV.

The detector is installed at the EuXFEL’s SQS instrument and has successfully been integrated into the NQS endstation, tested and subsequently commissioned in May 2019. The fully equipped NQS endstation is available for the European XFEL’s user community since June 2019 for investigations of the interaction of intense FEL pulses with clusters, nano-particles and small bio-molecules exploiting coherent diffraction imaging techniques. Since then, the pnCCD detector and the NQS endstation have already been successfully used by the scientific community for several experiments during user runs in the first and second half of 2019 at EuXFEL.

The detector system is integrated into the EuXFEL’s facility-wide control system Karabo, enabling the EuXFEL standardized control, online/offline data processing, on the fly data correction and data calibration. During the commissioning phase and first user experiments, stable operation with a noise level below 10 e^−^ ENC was demonstrated (see Table 1 ▸).

Further improvements of the performance of the detector are expected after the present sensor module has been replaced by a further optimized one. This major upgrade is planned for the second half of 2020. The new sensor module will provide an improved sensitivity for low-energy photons, enabled by the implementation of a thinner aluminium entrance window with a thickness of 60 nm in comparison with 150 nm of the present one. This results in a factor of 1.5 higher quantum efficiency at 0.25 keV. An extensive multi-energy calibration campaign of the pnCCD will follow this upgrade, with the main goals of enabling best possible sensitivity and the spectroscopic capabilities of the pnCCD with Fano-noise-limited energy resolution.

Further information about the SQS instrument, the NQS station, data processing/correction, and the pnCCD detector can be found on the EuXFEL’s webpages (SQS, 2020 ▸).

## Figures and Tables

**Table 1:** Detector performance parameters as achieved at the SQS experiment in comparison with detector parameters

	Achieved at SQS
Energy range	0.5–3 keV
Pixel size	75 µm × 75 µm
Position resolution	<10 µm
Sensor thickness	450 µm
Charge handling capacity	∼5 × 10^5^ e^−^ pixel^−1^
Sensitive area (both sensor halves)	7.7 cm × 7.7 cm
Dynamic range (low gain)	6000 photons @ 1 keV
RMS noise	10.5 e^−^ (gain 1, high gain)
	13.6 e^−^ (gain 1/16, low gain)
Single photon sensitivity	6.6σ at ≥250 eV
	13.2σ at 500 eV
Maximum frame rate	Up to 100 Hz
